# An RVFLNs ensemble modeling method integrating PCA and PSO: Application to yield prediction of Nongxiang Baijiu

**DOI:** 10.1371/journal.pone.0348784

**Published:** 2026-05-06

**Authors:** Qiang Han, Yibo Xu, Suyi Zhang, Qinwen Deng, Lan Deng, Liang Zhang, Hui Qin, Jie Zhao, Bo Liu

**Affiliations:** 1 Intelligence Perception and Control Key Laboratory of Sichuan Province, School of Automation and Information Engineering, Sichuan University of Science & Engineering, Yibin, Sichuan, China; 2 Luzhou Laojiao Co., Ltd., Luzhou, China; 3 Department of Automation, Tsinghua University, Beijing, China; 4 Solid-state Brewing Technology Innovation Center of Sichuan, Luzhou, China; China University of Geosciences, CHINA

## Abstract

To investigate the mapping relationship between key process parameters and Baijiu yield during the steaming and distillation process of Baijiu fermented material (SDP-BFM) and to optimize these parameters for enhanced production efficiency, a Random Vector Functional Link Networks (RVFLNs) ensemble modeling method integrating Principal Component Analysis (PCA) and Particle Swarm Optimization (PSO) is proposed for yield prediction of Nongxiang Baijiu. First, to improve computational efficiency and avoid multicollinearity, PCA is applied to reduce the dimensionality of the high-dimensional output matrix of RVFLNs, following data cleaning and feature selection. Second, a PSO algorithm is introduced to optimize both the number of hidden layer nodes in sub-learners and the weight combination strategy of the ensemble linear regression method, ultimately achieving Baijiu yield prediction modeling based on the PSO-P-ERVFLNs algorithm. Comparative experiments demonstrate that the optimization strategy introduced by PSO can enhance the prediction accuracy of the RVFLNs algorithm and alleviate overfitting. Moreover, the proposed algorithm exhibits better computational efficiency and higher estimation accuracy, enabling accurate prediction of Baijiu yield during the SDP-BFM.

## 1. Introduction

Chinese Baijiu, recognized as one of the world’s seven major distilled spirits, is a traditional liquor with a millennia-old heritage and unique fermentation craftsmanship. The production process of traditional solid-state fermented Baijiu involves three key stages: qu-making (including grinding materials, brick pressing, chamber placement, and regular turning), liquor making (mixing ingredients, steaming, spreading, adding qu, anaerobic pit fermentation, and distillation), followed by extended aging and expert blending [1,2]. In the Baijiu brewing process, the steaming and distillation process of Baijiu fermented material (SDP-BFM) is a critical step for extracting flavor compounds from the fermented grains [3–5]. This step largely determines the quality of the base liquor and the final distillation yield.

Therefore, developing a data-driven model to predict Baijiu yield during in the SDP-BFM is of great significance for exploring the relationship between key process parameters and production yield and for optimizing these parameters. However, most existing studies have focused on the impact of individual production indicators on Baijiu quality [6–8], with limited research on comprehensively analyzing all key factors influencing Baijiu yield during in the SDP-BFM [9].

Moreover, the SDP-BFM involves complex mechanisms, exhibiting strong nonlinearity and variable coupling [10]. Data-driven modeling methods do not require prior knowledge of these internal complexities and can construct predictive models solely through machine learning algorithms and industrial data processing [11], making such approaches a recent research hotspot in industrial process modeling [12–14]. For example, reference [15] used an artificial neural network to predict daily oil production based on operational parameters. Reference [16] developed a shale gas production forecast model using geological and engineering parameters. Reference [17] combined neuro-fuzzy inference with gene expression programming to construct a hybrid prediction model for electricity consumption, improving convergence speed and robustness while reducing computation time and model error. Given the effectiveness of these data-driven methods, building a yield prediction model for Baijiu represents a critical step toward full automation in Baijiu production.

In recent years, the Random Vector Functional-Link Network (RVFLNs) has gained popularity in industrial prediction due to its ability to handle complex nonlinear data, fast training speed, and strong generalization performance, effectively addressing issues such as long training time, sensitive hyperparameter tuning, and local minima in traditional neural networks [18–20]. For instance, reference [19] applied an improved RVFLNs with fixed hidden nodes to model molten iron quality, while reference [20] used an ensemble of RVFLNs with randomly generated hidden nodes within a certain range. Although these methods improved model performance to some extent, they neglected the optimization of the number of hidden nodes in RVFLNs, failing to control model complexity and potentially reducing prediction accuracy and generalization ability.

To address these issues, this study uses real-time operational data from an automated distillation system in a distillery along with Baijiu yield, aiming to solve two key problems: (1) extracting key variables relevant to the SDP-BFM through feature selection methods, and (2) Optimal selection of the number of hidden nodes in RVFLNs using intelligent optimization algorithms. First, to ensure model performance and reduce modeling complexity, feature selection is conducted by comparing the modeling performance of different feature selection methods, based on initial data cleaning and statistical analysis. Second, the number of hidden layer nodes is determined through comparative analysis of how modeling performance indicators vary with the number of nodes, thereby defining the sub-learners of the ensemble model. Finally, the intelligent optimization algorithm is employed to optimize both the hidden layer node number of sub-learners and the weight combination strategy of the ensemble linear regression method, ultimately establishing a Baijiu yield forecasting model based on the PSO-P-ERVFLNs algorithm. The aim is to accurately predict Baijiu yield during the SDP-BFM by exploring the complex mapping relationship between process parameters of the SDP-BFM and Baijiu yield.

## 2. Research methodology

### 2.1. RVFLNs base learners

RVFLNs were originally proposed by Pao and Takefuji [21] as a novel single-hidden-layer feedforward neural network architecture. The defining characteristic of RVFLNs lies in their randomized parameter initialization: both input weights and hidden layer biases are randomly assigned within specified bounds, while output weights are analytically determined through pseudo-inverse based least squares estimation. This unique design endows RVFLNs with several notable advantages, including rapid training speed, excellent generalization capability, and inherent suitability for robust modeling applications.

For N distinct arbitrary sample sets:


$$\overset{N}{\underset{i=1}{\left\{\left(x_{i},y_{i}\right)|x_{i}=[x_{i1},x_{i2},\cdots ,x_{in}]\right\}}}$$ (1)

A single-hidden-layer feedforward neural network with L hidden nodes and activation function φ(x) can be mathematically expressed as:


$$f_{wj}=\sum_{i=1}^{L}\beta_{i}\varphi_{i}\left(x_{i}\right)=\sum_{i=1}^{L}\beta_{i}\phi \left(\langle w_{i},x_{j}\rangle +b_{i}\right),j=1,2,\cdots ,N$$ (2)

where $\omega_{i}=[\omega_{i1},\omega_{i2},\cdots ,\omega_{in}{]}^{T}$ represents the weight matrix between the *i*-th hidden layer node and the input layer neurons, $\beta_{i}$ denotes the output weight matrix between the *i*-th hidden layer node and the output nodes, and $b_{i}$ is the bias term of the *i*-th hidden layer node.

Training traditional RVFLNs is equivalent to minimizing the following cost function:


$$C=\sum_{j=1}^{L}{\left(\sum_{i=1}^{L}\beta_{i}\phi \left(\langle w_{i},x_{j}\rangle +b_{i}\right)\right)}^{\text{2}}$$ (3)

### 2.2. PCA-based dimensionality reduction of RVFLNs hidden layer outputs

**Fig 1:**
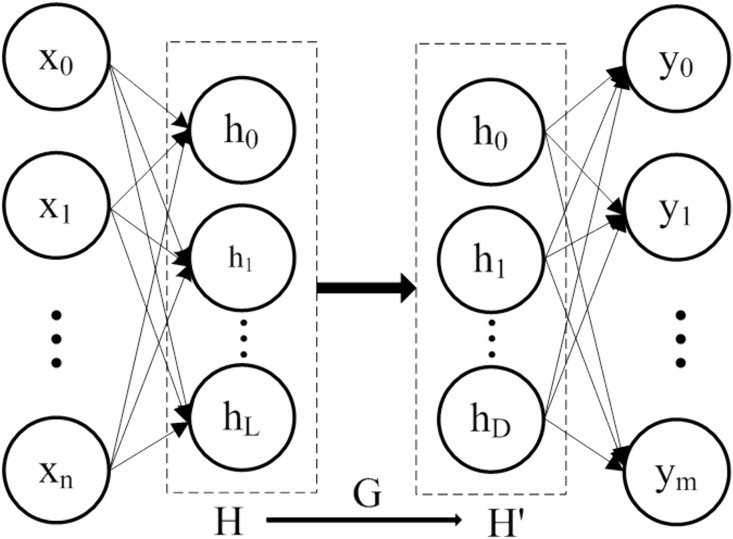
Architecture diagram of the principal component analysis (PCA)-enhanced RVFLNs algorithm (P-RVFLNs).

In RVFLNs, the randomness of input weights and hidden layer biases leads to multicollinearity issues in the hidden layer output matrix. This results in numerous redundant or low-contribution neurons, which not only complicates the network architecture but also reduces computational efficiency [22]. To address this issue, literature [23] applied PCA technology to perform dimensionality reduction on the hidden layer output matrix of RVFLNs, achieving significantly improved results. The structure of this P-RVFLNs algorithm is illustrated in Fig 1.

The PCA-based data dimensionality reduction process mainly involves the following steps: computing the covariance matrix, calculating eigenvalues and eigenvectors, determining the variance contribution rate and cumulative variance contribution rate of principal components to extract and interpret them. One primary objective of PCA is to reduce the number of variables while retaining maximal information from the original dataset. Typically, the top D principal components corresponding to eigenvalues with cumulative contribution rates between 85% and 95% are selected. As shown in Equation (4):


$$\left\{ \begin{array}{c} {h^{\prime }}_{\text{1}}=g_{11}h_{\text{1}}+g_{12}h_{\text{2}}+\cdots +g_{1L}h_{L}\\ {h^{\prime }}_{\text{1}}=g_{21}h_{\text{1}}+g_{22}h_{\text{2}}+\cdots +g_{2L}h_{L}\\ \vdots \\ {h^{\prime }}_{\text{1}}=g_{D1}h_{\text{1}}+g_{D2}h_{\text{2}}+\cdots +g_{DL}h_{L} \end{array}\right.$$ (4)

Rewriting the above in matrix form:


$$H^{\prime }=HG$$ (5)

where $H^{\prime }=\left[{h^{\prime }}_{\text{1}},{h^{\prime }}_{\text{2}},\cdots ,{h^{\prime }}_{D}\right]$, $G=[g_{ij}{]}_{D\times L}$.

### 2.3. Particle swarm optimization (PSO) algorithm

The Particle Swarm Optimization (PSO) algorithm is a heuristic global optimization technique. It operates by simulating a population of particles, each characterized by its own velocity and position in the search space. Through a combination of individual experience and group learning, the particles continuously adjust their movement, thereby progressively converging toward optimal solutions [24].

The implementation of the PSO algorithm begins with the initialization of a swarm of particles with random positions and velocities. For each particle, the fitness value of its current position is computed and used to update its personal best known position. Subsequently, both the individual particle’s best position and the global best position found by the entire population are utilized to update the velocity and position of each particle, guiding their trajectories toward promising regions of the search space.

The velocity update equation and the position update equation of the PSO algorithm are given in Equation (6) and Equation (7), respectively:


$$\overset{t+1}{\underset{i}{v}}=w.\overset{l}{\underset{i}{v}}+c_{\text{1}}.rand\left(\right).(pbest_{i}-\overset{t}{\underset{i}{x}})+c_{\text{2}}.rand\left(\right).(gbest_{i}-\overset{t}{\underset{i}{x}})$$ (6)

$$\overset{t+1}{\underset{i}{x}}=\overset{t}{\underset{i}{x}}+\overset{t+1}{\underset{i}{v}}$$ (7)

where $\overset{t+1}{\underset{i}{x}}$ denotes the position of the *i*-th particle at the *t*-th iteration, $\overset{t}{\underset{i}{v}}$ represen*t*s its velocity at the *t*-th iteration, pbest refers to the personal best position of *t*he *i*-th particle, and gbest indicates the global best position. w is the inertia weight, while $c_{\text{1}}$ and $c_{\text{2}}$ are the cognitive and social learning factors, respectively. rand() is a random number between 0 and 1. By continuously adjusting the positions and velocities of particles, the PSO algorithm can effectively search for the optimal solution to a problem [25].

### 2.4. Algorithm structure and implementation steps

**Fig 2:**
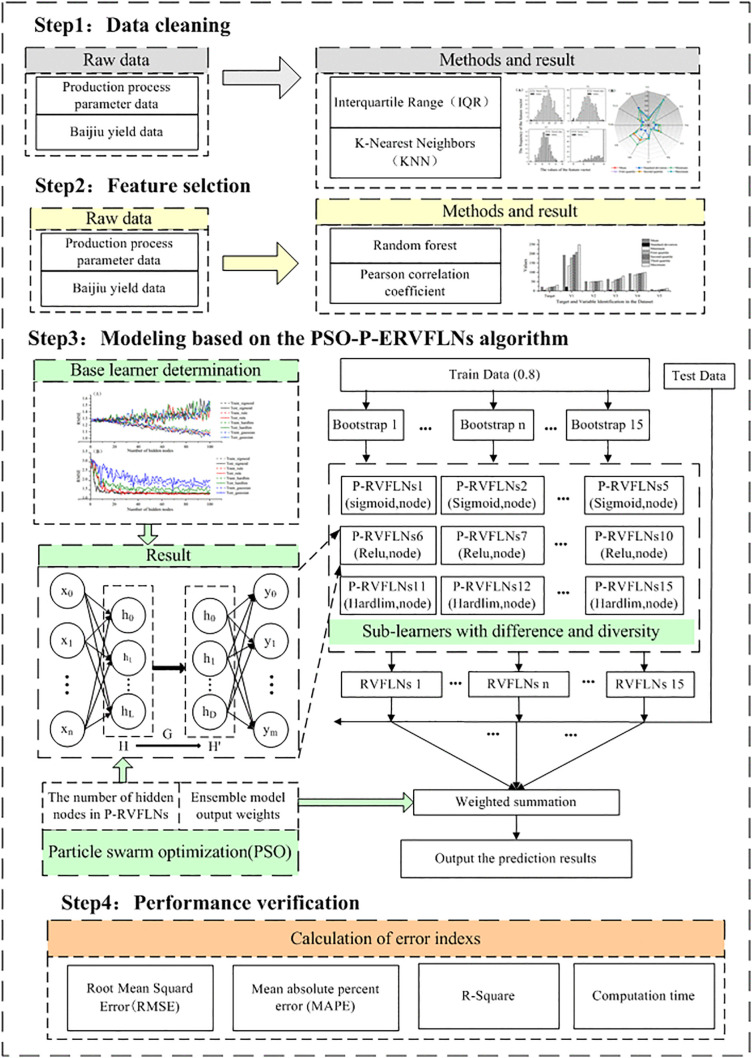
Structure of the PSO-P-ERVFLNs (an RVFLNs ensemble modeling method integrating PCA and PSO) Algorithm.

An ensemble model is a machine learning approach that combines the predictions of multiple base learners to significantly improve model performance, robustness, and generalization ability. Compared with a single learner model, ensemble models offer higher prediction accuracy and stronger generalization capability [26,27]. The most common ensemble algorithms include Bagging, Stacking, and Boosting. The core idea of Bagging is to generate multiple training subsets from the original dataset through bootstrap sampling, train a base learner on each subset, and finally integrate the regression results of these learners to obtain the final prediction. This approach can effectively enhance prediction accuracy and prevent overfitting. Therefore, to ensure the reliability and stability of liquor yield prediction during the SDP-BFM, an RVFLNs ensemble modeling method integrating PCA and PSO is proposed for predicting Baijiu yield in the SDP-BFM. The overall structure is shown in Fig 2.

Finally, the implementation steps of the PSO-P-ERVFLNs algorithm-based modeling are as follows:

1. Given the initial dataset $Z=\overset{N}{\underset{i=1}{\left\{\left(x_{i},y_{i}\right)|x_{i}=[x_{i1},x_{i2},\cdots ,x_{in}{]}^{T}\right\}}}$, perform data cleaning and statistical analysis, and conduct feature selection by comparing the modeling performance of different feature selection methods.
2. Determine the sub-learners P-RVFLNs of the ensemble model by analyzing how modeling performance metrics vary with the number of hidden layer nodes.
3. Divide the cleaned and feature-selected dataset $Z^{\prime }$ into a training set $D_{r}$ and a testing set $D_{e}$. Apply bootstrap sampling on $D_{r}$ to generate N training subsets $\left\{D^{\text{1}},D^{\text{2}},\cdots ,D^{N}\right\}$ (here, N = 15).
4. Train 15 P-RVFLNs-based sub-learners on the training subsets $\left\{D^{\text{1}},D^{\text{2}},\cdots ,D^{N}\right\}$. Each sub-learner is generated through perturbation of the base learner, with specific strategies as follows: five P-RVFLNs sub-learners using Sigmoid, ReLU, and Hardlim activation functions, respectively. The weights and biases of each sub-learner are randomly selected within the range [−1, 1], and the number of hidden layer nodes is optimized using the PSO algorithm.
5. Use all sub-learners to predict the testing set $D_{e}$. Optimize the weight combination strategy of the ensemble linear regression method with PSO, take the weighted sum of sub-learners’ predictions as the final result, and evaluate the model performance.

## 3. Experimental results and discussion

This study collected data on Baijiu production process parameters from an automated brewing workshop of a distillery in Luzhou City, Sichuan Province, during November and December 2024. Based on the brewing technology and the configuration of relevant instruments, 12 key process variables affecting Baijiu yield during the SDP-BFM were identified. The characteristics of the corresponding dataset are summarized in Table 1.

**Table 1:** Dataset Feature Descriptions.

Process Parameters	Feature Identification	Unit
Bottom-pot steam pressure	V1	KPa
Bottom-pot steam flow rate	V2	kg/h
Distillate collection flow rate	V3	L/min
Condenser temperature	V4	°C
Alcohol vapor pipeline temperature	V5	°C
Alcohol vapor pipeline pressure	V6	KPa
Steam regulating valve feedback	V7	NA
Cooling water inlet valve feedback	V8	NA
Steamer basket temperature	V9	°C
Steaming duration	V10	min
Loading machine temperature	V11	°C
Loading machine lifting height	V12	mm
Baijiu yield	Target	kg

NA: not available.

### 3.1. Data cleaning

**Fig 3:**
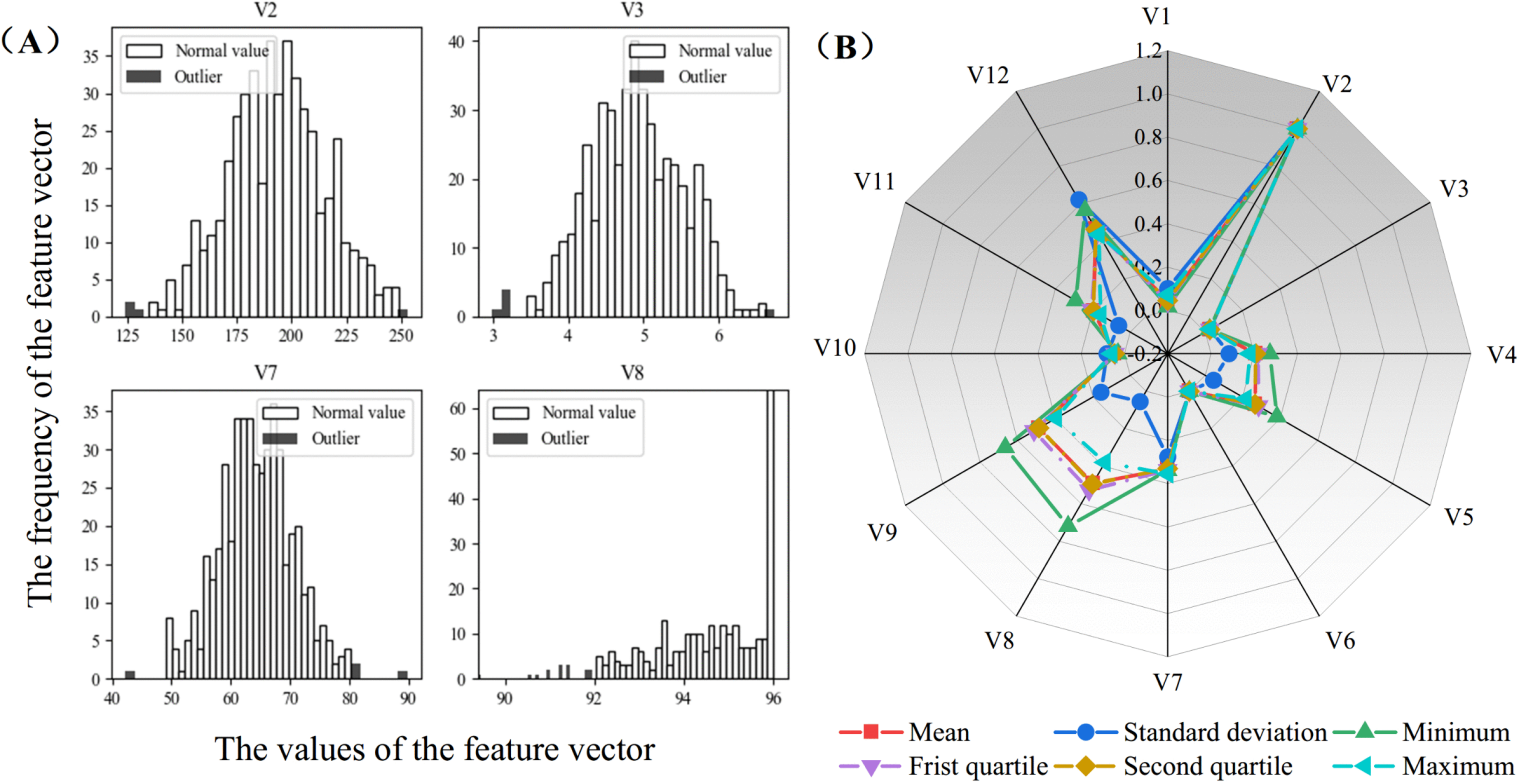
Data Processing Results. (A) presents histograms of four features—V2, V3, V7, and V8—that contained more captured outliers. As shown in **(A)**, the effectiveness of the IQR method in detecting data outliers can be more clearly and intuitively illustrated. (B) provides a clear statistical analysis of the dataset. These data processing methods improve the accuracy of data analysis and provide a reliable foundation for subsequent modeling.

Firstly, the collected data were segmented according to individual distillation batches based on the Baijiu brewing process, resulting in a total of 483 datasets comprising production process parameters and corresponding Baijiu yield values. Subsequently, the Interquartile Range (IQR) method was employed to detect outliers [28], identifying a total of 44 anomalous data points, which were visualized using histogram plots. Missing values were then imputed using the K-Nearest Neighbors algorithm. Finally, the statistical analysis results after outlier detection and Min–Max normalization are shown in Fig 3.

### 3.2. Optimal feature selection

The selection of feature variables is crucial for the predictive performance of the constructed model [29]. In this study, Pearson correlation analysis was first conducted to evaluate feature relevance. Features with correlation coefficients below 0.2 relative to the target variable were eliminated to exclude low-relevance variables, while those exhibiting inter-correlation above 0.8 were removed to mitigate multicollinearity. Subsequently, a Random Forest regression model optimized via grid search was employed to rank feature importance. The top features, equal in number to those retained after Pearson filtering, were selected as the final feature subset.

**Fig 4:**
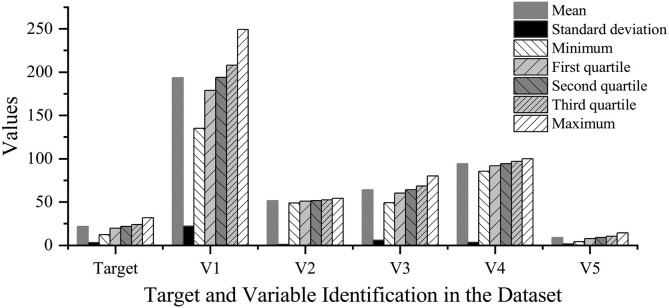
Histogram of Statistical Analysis of the Dataset after Feature Selection by the Random Forest Algorithm.

The feature sets obtained from the Pearson method, the Random Forest algorithm, as well as the full unselected feature set, were each used as input to individual RVFLNs base learners for modeling. The weights and biases of the hidden layer in each base learner were randomly initialized within the interval [−1, 1] following reference [30]. The number of hidden nodes was uniformly set to 20, and the sigmoid function was adopted as the activation function. The dataset was split into training and testing sets with a ratio of 8:2. The results of the feature optimization process are summarized in Table 2, and a statistical overview of the dataset after feature optimization using Random Forest is provided in Fig 4.

**Table 2:** Results of the Feature Optimization.

Methods	Feature Variables	Number of Variables	RMSE	R^2^
Pearson correlation analysis	V2、V9、V5、V7、V11	5	1.3526	0.8070
Random forest	V2、V9、V7、V5、V10	5	1.3198	0.8168
NA	V1、V2、V3、V4、V5、V6、V7、V8、V9、V10、V11、V12	12	1.5327	0.7529

NA: not available.

As shown in Table 2, the feature set selected by the Random Forest algorithm yielded the best performance, achieving the lowest Root Mean Square Error (RMSE) and the highest coefficient of determination (R²). Therefore, this feature set was chosen as the input for subsequent modeling. Compared with the unselected feature set, the results demonstrate that appropriate variable selection and screening are essential for constructing accurate and reliable prediction models.

### 3.3. Baijiu yield prediction experiments during the SDP-BFM

**Fig 5:**
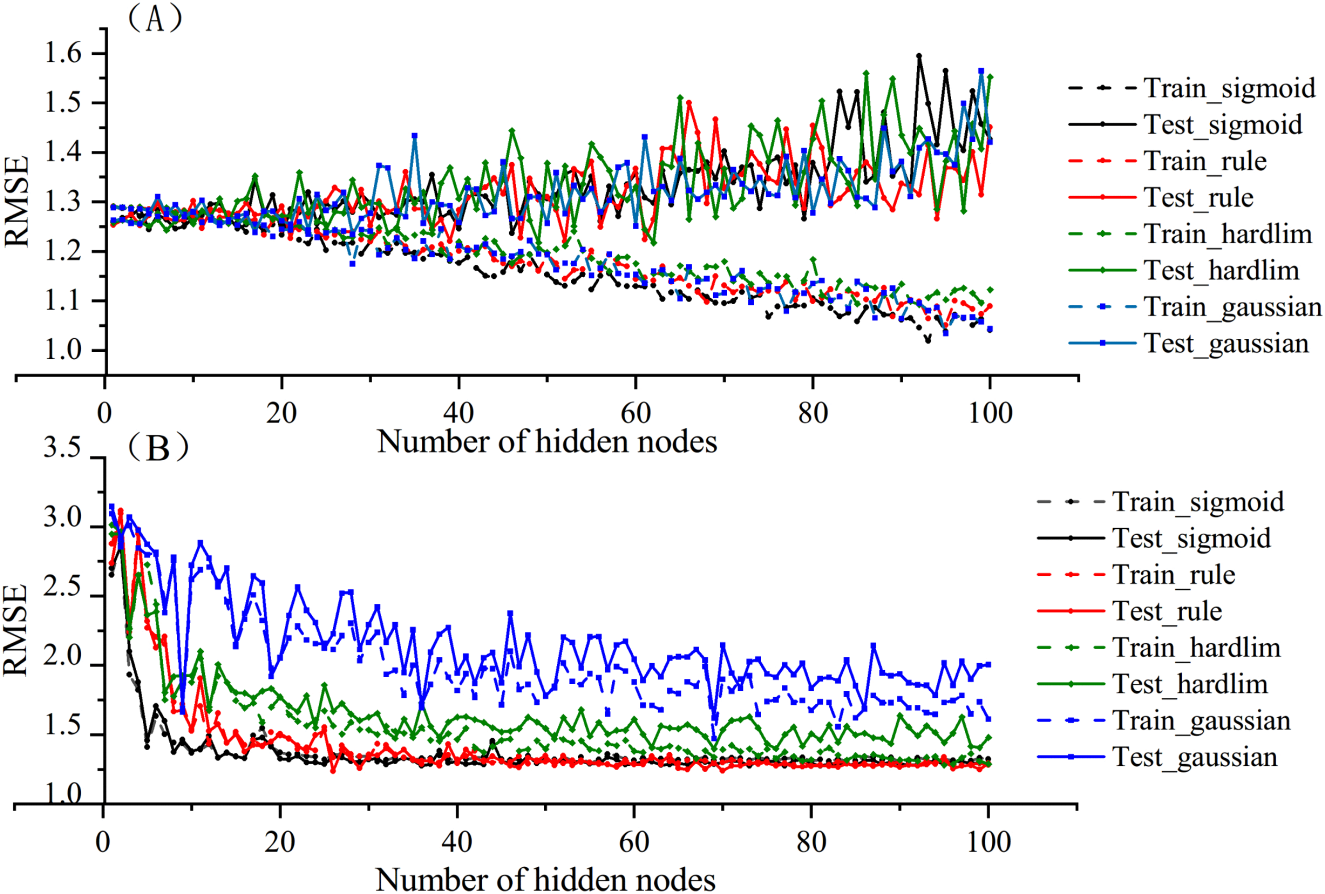
Sensitivity Analysis of Hidden Layer Node Count. Curve of RMSE versus Number of Hidden Layer Nodes for RVFLNs and P-RVFLNs Sub-learners with Different Activation Functions.

After data cleaning and feature selection, the proposed PSO-P-ERVFLNs algorithm is implemented based on bootstrap sampling for sample perturbation, with additional parameter perturbations applied to the base learners. The specific strategies are as follows: (1) the feature set selected by the random forest is used as the input of the subsequent model, with the training and testing sets divided at a ratio of 8:2; (2) the weights and biases of each sub-model’s hidden layer are randomly selected within the range of [−1, 1], following the approach in [30]; (3) this study investigates the changes in RMSE of the training and testing sets for both the RVFLNs and P-RVFLNs algorithms as the number of hidden layer nodes increases, in order to examine the algorithm’s ability to address overfitting, as shown in Fig 5.

As shown in Fig 5(A), with the increase in the number of hidden layer nodes, the RVFLNs algorithm exhibits overfitting: the RMSE of the training set shows a downward trend, while that of the testing set shows an upward trend. In contrast, Fig 5(B) illustrates that, except for the Gaussian activation function, the RMSEs of both the training and testing sets for the P-RVFLNs algorithm decrease and eventually stabilize as the number of hidden layer nodes increases. This improvement can be attributed to the PCA technique introduced in P-RVFLNs, which reduces the dimensionality of the high-dimensional hidden layer output matrix.

**Fig 6:**
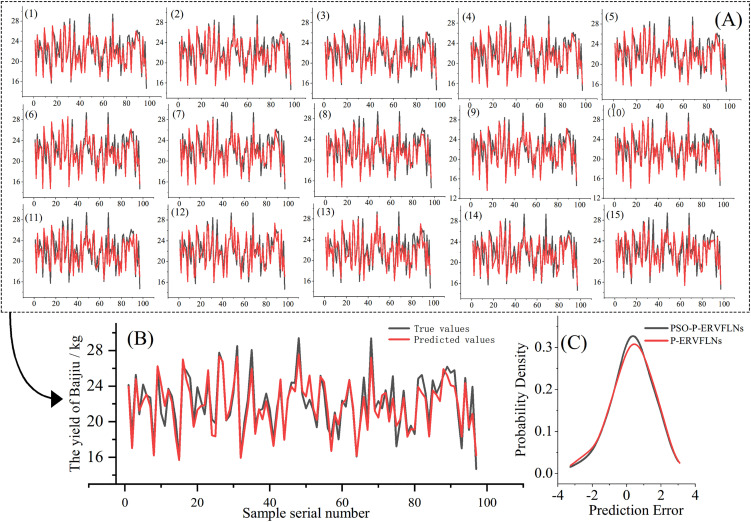
Modeling Effect Comparison Chart. **(A)** Performance of each sub-model. **(B)** Performance of the ensemble model. **(C)** Comparative analysis of model error curves.

Meanwhile, to avoid the influence of activation function selection on model performance, only P-RVFLNs sub-learners with Sigmoid, ReLU, and Hardlim activation functions were retained, with five sub-learners of each type, generating a total of 15 RVFLNs sub-learners. The particle swarm optimization (PSO) algorithm was then applied to optimize both the number of hidden layer nodes of the P-RVFLNs sub-learners and the weight combination strategy of the ensemble linear regression method, ultimately achieving Baijiu yield prediction modeling based on the PSO-P-ERVFLNs algorithm. Fig 6 presents the prediction performance of each P-RVFLNs sub-model and the ensemble model before PSO optimization.

Fig 6(A) shows the prediction performance of each P-ERVFLNs sub-model and the ensemble model after optimization with the particle swarm optimization algorithm. Fig 6(B) presents the prediction results of the ensemble model based on PSO-P-ERVFLNs. Fig 6(C) compares the probability density functions (PDFs) of prediction errors before and after optimization to verify the effectiveness of the proposed PSO-based optimization. From the error PDFs, it can be observed that the liquor yield prediction model optimized with the PSO algorithm achieves higher accuracy.

**Fig 7:**
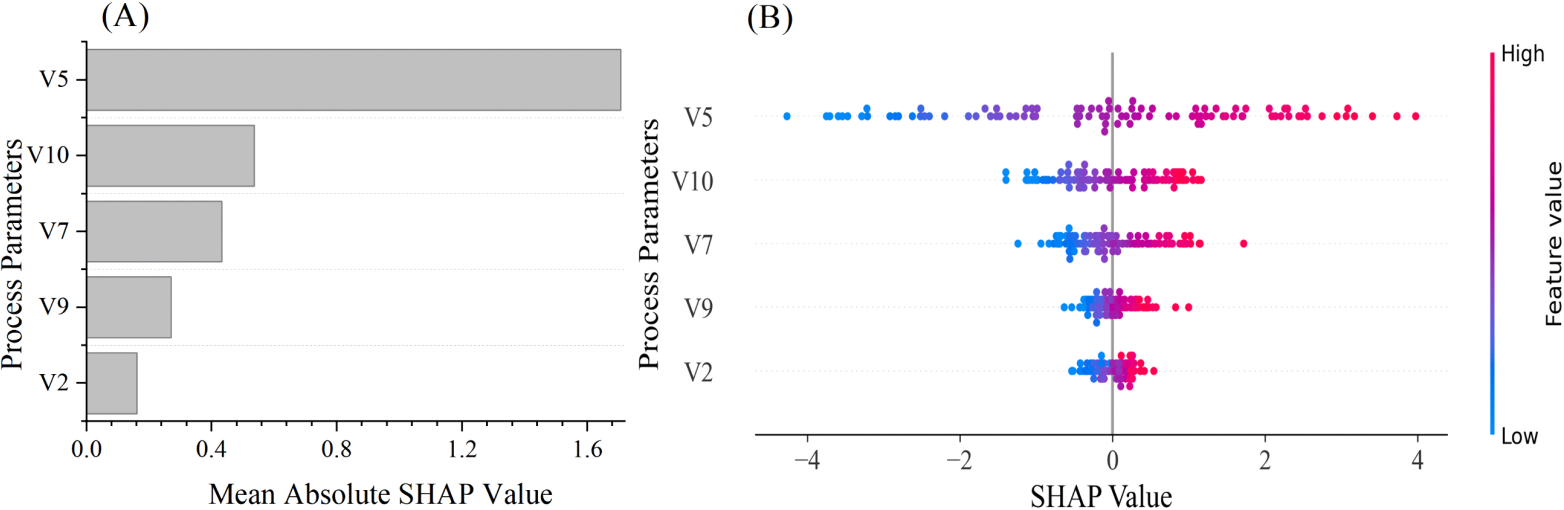
Results of the SHapley Additive exPlanations (SHAP) Model Analysis. **(A)** Reflects the overall impact intensity of each parameter on the model output; the higher the value, the greater the impact. **(B)** Shows the distribution of SHAP values for each parameter, indicating the direction and range of each parameter’s influence on the prediction results; the magnitude reflects the strength of the positive contribution.

Although the PSO-P-ERVFLNs model can establish the mapping relationship between process parameters and Baijiu yield, it lacks interpretability. Therefore, the SHapley Additive explanation (SHAP) interpretation model was applied for further analysis, and the specific results are shown in Fig 7. Fig 7(A) presents the feature importance bar chart, indicating the influence level of each parameter, while Fig 7(B) shows the SHAP scatter plot, illustrating the influence range and pattern of each parameter. As shown in Fig 7, during the SDP-BFM, variable V5 has the greatest impact on Baijiu yield, followed by V10, V7, V9, and V2.

Finally, to further enhance the robustness of the model and verify its generalization capability, a ten-fold cross-validation was conducted to evaluate the performance of the PSO-P-ERVFLNs model [31,32], and model sensitivity analysis was performed by adding Gaussian noise of different intensities to the test set. The results of the ten-fold cross-validation and sensitivity analysis are shown in Table 3.

**Table 3:** Results of Ten-Fold Cross-Validation and Sensitivity Analysis.

Methods	Gaussian Noise Intensity	Statistical Summary	Performance Evaluation Metrics		
			RMSE	MAPE	R^2^
PSO-P-ERVFLNs	NA	Mean	1.2611	4.59%	0.8270
		Standard Deviation	0.1526	0.47%	0.0466
	2.5%	NA	1.2499	4.5727%	0.8356
	5.0%		1.2489	4.5473%	0.8359
	10.0%		1.3100	4.7351%	0.8194
	15.0%		1.2442	4.6010%	0.8371
	20.0%		1.3459	4.8400%	0.8094

NA: not available.

As shown in Table 3, after applying ten-fold cross-validation, the RMSE has an average value of 1.2611 with a standard deviation of 0.1526, indicating small error fluctuations across the folds. The MAPE is 4.59%, with a standard deviation of less than 0.5%, suggesting that the model’s average relative error is very low and its stability is high. The average coefficient of determination is approximately 0.83, with a standard deviation of only 0.0466, demonstrating good generalization consistency of the model.

In addition, under different levels of Gaussian noise intensity, the variations in model performance metrics remain minimal, with no significant performance fluctuations observed. This indicates that the model’s prediction results are stable and not sensitive to input perturbations. Therefore, the PSO-P-ERVFLNs model exhibits strong robustness and noise resistance, making it suitable for liquor yield prediction tasks that involve certain measurement errors.

### 3.4. Comparative experiments

**Fig 8:**
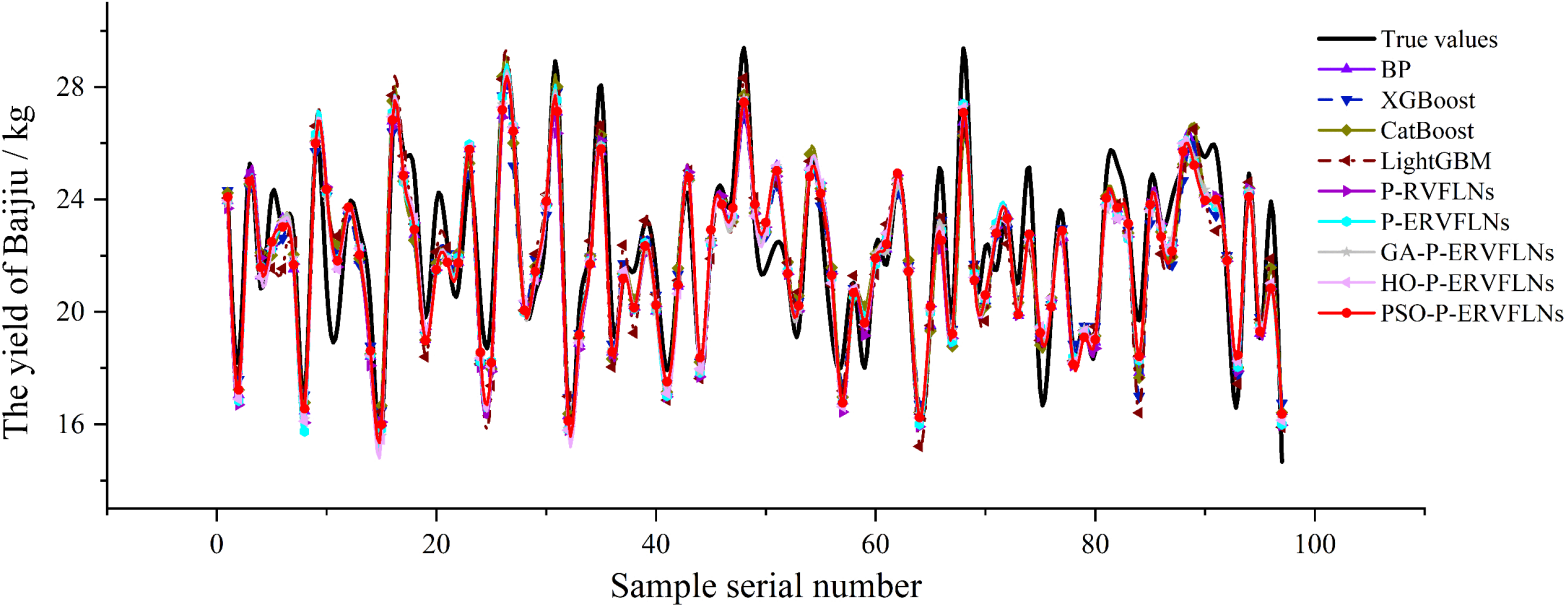
Prediction Results of Baijiu Yield Modeling with Different Algorithms.

To further verify the effectiveness and superiority of the proposed algorithm in Baijiu yield prediction during the SDP-BFM, the PSO-P-ERVFLNs algorithm is compared with the P-RVFLNs algorithm, P-ERVFLNs algorithm, GA-P-ERVFLNs algorithm, HO-P-ERVFLNs algorithm, as well as the Back Propagation Neural Network (BPNN), LightGBM algorithm, CatBoost algorithm and XGBoost algorithm, using the same dataset for predictive experiments. Root Mean Squared Error (RMSE), Mean Absolute Percentage Error (MAPE), and the coefficient of determination (R²) are employed to evaluate the prediction performance of models constructed by different algorithms. The results of the comparative experiments are reported as the average values of five independent runs. The parameter settings and evaluation results of the algorithms are summarized in Table 4. The Baijiu yield prediction results modeled by different algorithms are illustrated in Fig 8.

**Table 4:** Details of Model Parameters and Performance Evaluation Results for Baijiu Yield Prediction with Different Algorithms.

Methods	Parameter Details		Performance Evaluation Metrics			
	Activation Function	Hidden Layer Nodes	RMSE	MAPE	R^2^	Computation time
P-RVFLNs	Sigmoid	80	1.2943	4.671%	0.8237	0.01562
P-ERVFLNs	Sigmoid	Ranking Selection	1.2641	4.593%	0.8319	0.01565
	Relu					
	Headlim					
GA-P-ERVFLNs	Sigmoid	GA-based Optimization Selection	1.2398	4.483%	0.8383	0.03120
	Relu					
	Headlim					
HO-P-ERVFLNs	Sigmoid	HO-based Optimization Selection	1.2444	4.593%	0.8319	0.03214
	Relu					
	Headlim					
PSO-P-ERVFLNs	Sigmoid	PSO-based Optimization Selection	1.2206	4.441%	0.8432	0.032124
	Relu					
	Headlim					
BPNN	Sigmoid	17	1.2994	4.673%	0.8265	0.26560
XGBoost	NA	NA	1.2966	4.670%	0.8231	0.00598
LightGBM	NA	NA	1.3529	4.9431%	0.8074	0.00900
CatBoost	NA	NA	1.2990	4.7560%	0.8224	0.01560

NA: not available.

The number of hidden layer nodes in the P-ERVFLNs algorithm is determined in Section 3.3, based on the RMSE variation curves of RVFLNs and P-RVFLNs sub-learners with the same activation function. Specifically, the top five hidden layer node settings with the lowest RMSE values of the P-RVFLNs sub-learners are selected. For the GA-P-ERVFLNs, HO-P-ERVFLNs, and PSO-P-ERVFLNs algorithms, the number of hidden layer nodes is optimized within the range [1, 200] using the GA, HO, and PSO algorithms, respectively. The hyperparameters of XGBoost, LightGBM and CatBoost are optimized using grid search.

As shown in Table 4 and Fig 8, by comparing the PSO-P-ERVFLNs algorithm with BPNN, XGBoost, LightGBM, and CatBoost, it can be observed that the model constructed by PSO-P-ERVFLNs demonstrates better computational efficiency and higher estimation accuracy. Furthermore, by comparing the RMSE performance metrics of the P-RVFLNs, P-ERVFLNs, GA-P-ERVFLNs, HO-P-ERVFLNs, and PSO-P-ERVFLNs algorithms, it can be observed that incorporating PCA for output matrix dimensionality reduction, together with applying the PSO algorithm to optimize both the hidden layer node number of P-RVFLNs sub-learners and the weight combination strategy of ensemble linear regression, can effectively improve the prediction accuracy of the RVFLNs algorithm and mitigate overfitting issues.

## 4. Conclusion

In this study, an RVFLNs ensemble modeling method integrating PCA and PSO is proposed to establish a Baijiu yield prediction model for the SDP-BFM. First, the feature variables selected by the Pearson correlation coefficient and random forest algorithm, as well as those without selection, are separately used as inputs to RVFLNs sub-learners for modeling. By comparing the RMSE performance metrics of the models, it is found that the feature set selected by the random forest yields the best results. Second, through comparative analysis of modeling performance metrics with varying numbers of hidden layer nodes, the sub-learners of the ensemble are determined to be P-RVFLNs. Then, on this basis, the particle swarm PSO algorithm is employed to optimize both the hidden layer node number of sub-learners and the weight combination strategy of ensemble linear regression, ultimately realizing Baijiu yield prediction modeling based on the PSO-P-ERVFLNs algorithm.

Finally, by comparing the RMSE performance metrics of the P-RVFLNs, P-ERVFLNs, GA-P-ERVFLNs, HO-P-ERVFLNs, and PSO-P-ERVFLNs algorithms, it is shown that incorporating PCA for dimensionality reduction of the output matrix, along with PSO for optimizing the hidden layer node number of P-RVFLNs sub-learners and the weight combination strategy of ensemble linear regression, can indeed improve the prediction accuracy of the RVFLNs algorithm and mitigate overfitting to a certain extent. Moreover, the model constructed by the PSO-P-ERVFLNs algorithm demonstrates better computational efficiency and higher estimation accuracy, enabling accurate prediction of Baijiu yield during the SDP-BFM. Nevertheless, although the proposed PSO-P-ERVFLNs algorithm performs well on the experimental dataset, due to limitations in current Baijiu production processes and data collection techniques, it has not been validated on severely nonlinear data. Therefore, its capability to handle highly dynamic or strongly nonlinear data may be limited.

## Supporting information

S1 File Raw Data. (CSV)
